# Online shopping motivations and behavioral heterogeneity of digital nomads in livestream e-commerce: the moderating role of celebrity worship

**DOI:** 10.3389/fpsyg.2026.1823174

**Published:** 2026-05-20

**Authors:** Bingjie Sun, Wenjing Ke

**Affiliations:** 1 School of Business Administration, Xinjiang University of Science & Technology, Korla, China; 2 College of Business, Wuyi University, Wuyishan, China

**Keywords:** agricultural livestream e-commerce, characteristic forest-fruit industry, digital nomad online shopping, influencer worship, online shopping behavior, online shopping motivation, parasocial relationships

## Abstract

**Introduction:**

Digital nomads constitute a heterogeneous and rapidly growing consumer segment in livestream e-commerce, yet their motivational structure and the conditional role of celebrity worship in shaping their behavior remain underexplored, particularly in the context of digital transformation of China's characteristic forest-fruit industries.

**Methods:**

This study employs a three-wave mixed-methods design combining in-depth interviews, qualitative text mining, and quantitative questionnaire surveys with 1,255 Chinese digital nomads (including a third-wave subsample of 250 from Southern Xinjiang). Factor analysis, K-means clustering, hierarchical regression, and robustness checks were conducted to identify motivational dimensions, classify subgroups, and test moderation effects.

**Results:**

Six motivational dimensions and 21 sub-indicators were identified: understanding influencer recommendations, seeking social interaction, achieving shopping convenience, building identity recognition, obtaining entertainment experiences, and participating in online communities. Cluster analysis revealed four motivational types (social interaction, comprehensive identification, practical convenience, identity pursuit). Celebrity worship significantly moderated key motivation-behavior relationships, positively moderating identity recognition's effect on satisfaction and repurchase intention, and negatively moderating entertainment seeking's effect on satisfaction. Findings replicated in the Southern Xinjiang subsample.

**Discussion:**

This study addresses gaps in dimensional motivation research, enriches understanding of within-group heterogeneity, provides a validated measurement tool, and offers practical guidance for platforms and merchants in optimizing marketing strategies. The findings provide a transferable consumer-side framework for livestream-channel strategies in the digital transformation of characteristic forest-fruit and specialty agricultural industries.

## 1 Introduction

Against the backdrop of the rise of the gig economy and remote work, flexible employment personnel, as the main force of new employment forms, number 200 million, accounting for 14.2% of the total population, according to data from the Statistical and Monitoring Report on New Employment Forms published by the Ministry of Human Resources and Social Security (Lovinska et al., 2020). Among them, about 84 million rely on internet platforms for employment, making up 42% of flexible workers (Rani and Furrer, 2020). Issues related to understanding, guiding, and serving this group are crucial to employment stability and social harmony (Ho and Chan, 2022). China's digital nomad community, influenced by relatively flexible working styles and weaker geographical constraints, tends to lead more nomadic, atomized lives, characterized by weaker social connections and professional belonging, as well as stronger spiritual needs (Li and Zhong, 2025). The enhancement of digital skills, disposable income, and leisure demands has also made “new digital nomads” one of the active and research-worthy groups in the consumer market (Malinen, 2015). National livestreaming e-commerce data show that livestreaming online shopping users account for more than 45% of internet users, and the livestreaming e-commerce market size exceeded 4.9 trillion yuan in 2023 (Wan et al., 2024).

In parallel, the digital transformation of China's characteristic agricultural and forest-fruit industries has increasingly shifted its critical bottleneck from upstream production and cold-chain infrastructure toward the consumer-facing end, where livestream e-commerce, influencer-led recommendation, and community-based consumption are reshaping how specialty products such as Xinjiang apples, Korla pears, and Xinjiang jujubes reach mobile, digitally embedded consumer groups (Jin and Zhang, 2025; Dong et al., 2022; Zhao, 2025). Among these groups, digital nomads represent a theoretically important segment: they are heavy livestream users, hold above-average discretionary income, and their consumption logic is closely tied to identity construction and parasocial community participation. These three features directly condition whether market-side digitalization can translate supply-side digital investments into durable demand (Huo et al., 2025). Clarifying the motivational dimensions and heterogeneity of this group therefore provides an empirical foundation not only for theorizing digital-nomad consumption, but also for designing consumer-side strategies within broader characteristic forest-fruit industry digital transformation programs, particularly in regions such as Southern Xinjiang where geographically indicated products depend heavily on livestream channels to reach distant consumer markets.

Notably, livestreaming online shopping by digital nomads is an emerging phenomenon in recent consumer behavior research. In the postmodern era, the breakdown of traditional social bonds and stable career paths has led to a sense of loss of belonging and identity anxiety among digital nomads, creating a psychological vacuum and urgent social needs that require new outlets (Chen et al., 2023). Within this context, celebrity worship—defined as fans' emotional attachment, psychological identification, and value internalization toward internet celebrities (Brooks, 2021)—serves as a critical psychological mechanism. The instant interaction, community atmosphere, and construction of internet celebrity personified IP in livestreaming e-commerce provide digital nomads with a platform for emotional connection and identity projection, with celebrity worship acting as the cognitive and affective bridge that transforms passive viewing into active para-social participation (Andino-Frydman, 2023). Compared to traditional online shoppers, digital nomads who engage in livestreaming shopping exhibit stronger exploratory intentions, greater emotional involvement, and more pronounced demands for community belonging. Relevant survey data show that digital nomads are high-value livestreaming users, with 35.7% spending over 2,000 yuan per month, far exceeding the spending of other online shopping groups (Gu et al., 2025). It is not only about browsing content related to celebrities and internet influencers in fields such as shopping, entertainment, and lifestyle, but also about actively participating in influencer fan groups, topic discussions, and online activities—an attempt by digital nomads to build stable social connections in their mobile lives to meet their needs for belonging and identity, with celebrity worship intensifying these participatory behaviors (Wang et al., 2022).

However, due to mainstream society's long-standing perception of digital nomadism as an informal and unstable form of employment (Jiwasiddi et al., 2024), the consumption behavior of digital nomads, along with their underlying psychological motivations, emotional needs, and social functions, has not received sufficient attention (Misra and Stokols, 2012). Furthermore, Chinese traditional culture emphasizes “attachment to one's native land and reluctance to move” and “establishing a family through industry,” which shape societal expectations for stable careers and fixed residences (Irimiás, 2023). Social public opinion often describes digital nomads' work methods and behaviors through negative stereotypes, such as “not sticking to one's proper job” and “loafing around” (Bonneau et al., 2023). Consequently, digital nomads' high involvement and emotional investment in livestreamed online shopping are frequently viewed as inconsistent with the image of a rational consumer, being labeled as blind idolization of internet celebrities or “irrational” (Gu et al., 2025). Relevant studies have also overlooked the uniqueness of digital nomads, instead focusing more on ordinary young internet users and interpreting livestreamed online shopping behavior as a simple manifestation of internet celebrity idolization or “herd effect” (Behrendt et al., 2019; Wu and Wilkes, 2018). Although a few studies have recognized the positive significance of digital nomads' participation in livestreamed online shopping activities, such as promoting consumption, alleviating loneliness, and enhancing life satisfaction (Luo et al., 2025), insufficient attention has been paid to the dimensional structure and measurement of their motivations. Critically, existing research has neither systematically incorporated celebrity worship as a core variable into the motivation measurement of digital nomad online shoppers, nor clarified whether and how different levels of celebrity worship moderate the relationship between specific motivations (e.g., identity building, social interaction, entertainment seeking) and behavioral outcomes (e.g., satisfaction, repurchase intention). The behavioral characteristic differences within this group remain unclear.

In summary, although the scale of digital nomads livestreamed online shopping is large and their characteristics are distinct, given their marginalized and heterogeneous survival status, measuring their motivations and behaviors remains a theoretical black box (Wilkesmann and Bassyiouny, 2025). Specifically, some critical questions, such as “What are the motivational dimensions of digital nomads?”, “Is there heterogeneity within the digital nomad group?”, “What psychological and behavioral characteristics do they exhibit in livestreamed online shopping?”, and “How does celebrity worship moderate the relationship between different motivational types and consumption behaviors?” urgently require rigorous scientific explanations from academia (Misra and Stokols, 2012). In view of this, this study focuses on digital nomads participating in livestreamed online shopping activities, identifies the motivational dimensions of digital nomads, classifies and characterizes them accordingly, and empirically examines the moderating role of celebrity worship in the motivation-behavior relationship. This provides a scientific measurement tool for subsequent empirical studies on digital nomads; at the same time, it also helps to promote society's attention and understanding of digital nomads, and offers decision-making references for relevant platform enterprises and service institutions to position niche markets and optimize products and services. To address these gaps, this study first identifies the motivational dimensions of digital nomad online shoppers and then classifies them into distinct subgroups based on their motivation patterns. Specifically, we anticipate that digital nomads may exhibit heterogeneous motivational structures—such as those prioritizing social interaction, practical convenience, identity pursuit, or comprehensive engagement—which will be empirically derived through cluster analysis. This classification approach allows for a more nuanced understanding of within-group differences. Concretely, our empirical analysis yields four motivational subgroups—the social interaction type, the comprehensive identification type, the practical convenience type, and the identity pursuit type—whose behavioral profiles and responsiveness to celebrity worship differ systematically. We further examine how celebrity worship, as an active psychological mechanism rather than a mere background characteristic, moderates the relationships between specific motivational dimensions (identity recognition, community participation, entertainment seeking) and downstream outcomes (satisfaction, repurchase intention, recommendation willingness). This dual focus on within-group heterogeneity and the moderating role of celebrity worship forms the organizing logic of the paper and connects the motivational dimensions identified in Sections 4.1 and 4.2, the four-cluster taxonomy developed in Section 4.5, and the moderation analysis reported in Section 4.6.

## 2 Literature review and hypotheses development

### 2.1 Celebrity worship and online shopping by digital nomads

The concept of “celebrity worship” originated from early discussions in social media research and fan culture. Later, in communication studies and sociology, it generally refers to the public's admiration for internet celebrities or their following of the lifestyles and values advocated by specific online opinion leaders (He and Liu, 2024). In the context of consumer society and media culture, the “internet celebrity economy” has come to be associated with the attention economy and emotional consumption (Qiu, 2025). Internet celebrities provide references for their fans' consumption decisions, aesthetic orientations, and identity recognition. Internet celebrity worship refers to phenomena such as fans' emotional attachment, psychological identification, attitudes, behaviors, consumption, and even internalization of values toward internet celebrities (Brooks, 2021), and it serves as the core of an emerging social psychological phenomenon and youth subcultural consumption practices (Zhang et al., 2025). Earlier studies identified celebrity worship as a unique type of fan relationship, which has been explored in fields such as research on social media influencers and consumer behavior (Zsila et al., 2018), as well as in celebrity worship and brand relationalization (Sansone and Sansone, 2014). It is worth noting that, because internet celebrity worship and celebrity worship are based on different relationship forms across media ecosystems, they differ in their motivations and behavioral manifestations (Chen et al., 2023). However, in comparison, celebrity worship emphasizes more on the core of a sense of distance from traditional stars and one-way admiration, focusing on idealized projection and fantasy (Zhang et al., 2025); while internet celebrity worship features higher and stronger interactive intimacy, daily content, and the accessibility shaped thereby, involving deeper and more daily relationships such as imitation, interaction, and community participation (He and Liu, 2024). While the literature distinguishes these two constructs, they share a common psychological core, namely emotional attachment to and identification with media figures (McCutcheon et al., 2002). For conceptual consistency and alignment with the dominant usage in the empirical psychology literature on which our measurement instrument is based, this paper hereafter adopts the unified term “celebrity worship” to refer to both traditional and internet-mediated forms, while acknowledging that our empirical focus lies on the internet-mediated form operative in livestream e-commerce contexts.

The academic community abroad has initially explored digital nomads' online shopping as a participatory behavior grounded in community identity and a lifestyle aspiration. Relevant studies have extensively examined the impact of celebrity worship on the formation of consumption attitudes (Zsila et al., 2018), purchase intention (Behrendt et al., 2019), and brand loyalty (Qiu, 2025). Additionally, the authenticity and credibility of influencers have been empirically supported in their roles in promoting consumer trust and willingness to participate, driving purchasing decisions and word-of-mouth dissemination (Zhao and Wu, 2021). In contrast, domestic research has focused more on the consumption behavior of Generation Z and on influencer livestreamed sales (Brooks, 2021) or on their behavioral characteristics and influencing factors from the perspectives of generational differences or motivation classification (McCutcheon et al., 2002). Research on the motivation structure and behavioral patterns of this specific group of digital nomads' online shopping is limited (McCutcheon et al., 2002). A few studies that focus on the consumption psychology and behavior of digital nomads place them within the context of a broader online consumer group or “new professional group” for exploration, but lack targeted, in-depth analysis.

In parallel, a growing body of research on agricultural livestream e-commerce has identified livestream selling as a primary market-side channel linking producers in remote regions to digitally dispersed consumer segments, and has begun to document its role as a driver of rural revitalization and characteristic-industry digital transformation (Dong et al., 2022; Zhao, 2025; Duan, 2023). Empirical studies in this stream have examined how streamer credibility, social presence, telepresence, and perceived informational quality shape consumer trust and purchase intention for green and geographically indicated agricultural products, including explicit examples drawn from Xinjiang specialty fruit livestreaming (Jin and Zhang, 2025; Huo et al., 2025). Notwithstanding these contributions, this literature has focused predominantly on supply-side actors—growers, new farmer streamers, cooperatives, platform operators, and cold-chain logistics providers—and on single-variable stimulus–response mechanisms at the point of purchase. The motivational structure of the mobile digital consumer segments that actually sustain livestream channels, and the within-segment heterogeneity that conditions how different motivational profiles respond to influencer-led content, remain largely unexamined. Integrating insights from digital-nomad consumption research with the emerging agenda on consumer-end digitalization of characteristic agricultural products is therefore both theoretically warranted and practically timely, especially for forest-fruit producing regions such as Southern Xinjiang whose market-end digitalization depends on sustained engagement from distant urban consumers.

### 2.2 Motivations of digital nomad online shoppers

The academic community has not yet reached a consensus on the definition and core characteristics of digital nomads. Early technological optimism and some social commentaries, as well as definitions of groups such as remote workers or global travelers, have identified people who work remotely using digital technology or conduct professional activities while living and moving globally as digital nomads (Miguel et al., 2025). However, at the level of empirical operation and quantitative research, most studies still use professional attributes and mobility (such as geographical independence, working Internet) or measurement of self-identification as “digital nomads” as screening criteria for digital nomad online shopping. Motivation, as the intrinsic psychological needs and behavioral drive behind online shopping, has been explored by foreign researchers in terms of the motivational dimensions of digital nomad online shoppers since the early days of the Internet's popularization (Nash et al., 2018). In recent years, online shopping behaviors driven by influencer economy have received more attention from academia, covering topics such as the role of social identity and conformity psychology, the impact of emotional value (seeking entertainment and emotional connection), and the influence of influencer traits and content quality on consumer trust and purchase intention (Ehn et al., 2022). To clarify the internal structure and dimensions of motivation among digital nomad online shoppers, the “Push-Pull Theory” has been applied widely (Prester et al., 2023). Relevant studies suggest that push factors include the desire to escape the constraints of traditional office work and pursue work autonomy, a flexible lifestyle, working while traveling, or living at a low cost; pull factors encompass high-quality network facilities and livable environments in the destination, as well as a better work-life balance. In addition, some researchers argue that the motivations of digital nomad online shoppers are not merely about functional and practical satisfaction, but are also influenced by the idealized lifestyle and identity constructed by influencers, a sense of belonging and interactive support from online communities (including fan groups), and collective consumption culture (Zhao and Wu, 2021). Motivations should be viewed as a dynamic construction process. Compared to the motivation structure of traditional online shoppers, digital nomad online shoppers, in addition to pursuing universal motivations such as product cost-effectiveness, shopping convenience, and service reliability, are deeply driven by their desire for geographical freedom, temporal autonomy, and a lifestyle (Zhao and Wu, 2021). They also significantly emphasize the lifestyle symbolized by recommendations, the social currency attributes of consumption behavior based on community interaction and content co-creation, resonance with brand stories and values, and self-expression and identity construction through consumption (Miguel et al., 2025). Relevant theoretical perspectives provide a helpful framework for understanding the motivations of digital nomad online shoppers (Bahri, 2024). However, there are shortcomings: research on internal motivational differences within the group of digital nomad online shoppers, as well as the mechanisms by which different types of motivation influence their consumption behavior patterns, remains insufficiently addressed by academia and industry (Gupta et al., 2024). The emerging phenomenon of digital nomad online shopping, along with its complex behaviors and profound generational and cultural drivers, has not received adequate attention (Simeli et al., 2025). Currently, there is a lack of effective measurement scales and research tools to assess the interactive and emotional motivations that digital nomad online shoppers rely on during the process, such as through influencer content and fan communities (Hannonen, 2020). Therefore, it is necessary to deeply explore the multidimensional structure and dynamic evolution of their motivations to enhance theoretical understanding and practical insights into the behavioral logic of digital nomad online shoppers.

### 2.3 Types and behavioral characteristics of digital nomad online shoppers

Digital nomad online shoppers exhibit significant differences in mobility characteristics, a light-asset lifestyle, and value orientations, which, in turn, lead to unique consumption scenarios, online shopping choices, and behavioral preferences (Prester et al., 2023). Studies have shown that digital nomads who value experiences and freedom place greater value on products and are more critical of their practical and emotional value (Bonneau et al., 2023). Therefore, they pay particular attention to whether commodities offer high performance at a high cost and spiritual compatibility, and they also rely more on social recommendations and focus on real feedback in their purchasing decisions and brand interactions (Atanasova, 2021). Digital natives, as the main group of online shoppers, possess proficient digital skills and a habit of social comparison, valuing the role of online communities in discovering products and validating decisions (Bahri, 2024). In contrast, digital immigrants, due to their reliance on tangible product perception and traditional habits, prefer offline channels and are more cautious about online shopping, showing clear channel preferences in purchasing fresh products and high-value goods. The academic community abroad has already produced a wealth of research on the behavior patterns and influencing factors of general online shoppers, including multi-channel shopping and time perception segmentation. Among these, smart solo shoppers, as a typical type of online shoppers, provide insights into understanding solo shopping. They are characterized by forming an offline shopping burden sense and an online shopping creation sense, based on time-value assessment, valuing the correctness of the first purchase, and being influenced by individualistic culture (Chevtaeva and Denizci-Guillet, 2021). Additionally, perspectives such as omni-channel behavior, social commerce drivers, experiential consumption shift, and values-based consumption have been explored. In the context of localized research, researchers have also explored domestic digital nomads as a cultural phenomenon, a new form of work organization, a new form of economic activity, and community builders (Yu et al., 2025). However, due to fundamental differences in lifestyle and varying levels of intrinsic motivation, theoretical frameworks and research findings based on fixed residents are insufficient to explain or predict the consumption logic and behavioral patterns of digital nomad online shoppers, who are more mobile and have values deeply rooted in experience pursuit and self-actualization (De Loryn, 2022). To address the academic gap in research on digital nomad online shoppers, this study aims to explore their unique consumption logic, decision-making mechanisms, and behavioral patterns, and to identify paths for experience creation, participation processes, and value realization through their behaviors (Prester et al., 2023).

### 2.4 The moderating role of celebrity worship

Drawing on Parasocial Relationship Theory (Horton and Wohl, 1956), we argue that celebrity worship is not merely a background characteristic but an active psychological mechanism that moderates the relationship between online shopping motivations and behavioral outcomes. Specifically, for digital nomads with higher levels of celebrity worship, the effects of identity-building motivation and community participation motivation on satisfaction and behavioral intentions are likely to be amplified, as these individuals derive greater emotional and social value from parasocial interactions. Conversely, for those with lower levels of celebrity worship, utilitarian motivations (e.g., shopping convenience, understanding recommendations) may play a more dominant role.

Based on this reasoning, we propose the following hypotheses:

H1: The six motivational dimensions (understanding influencer recommendations, achieving shopping convenience, seeking social interaction, building identity recognition, obtaining entertainment experiences, and participating in online communities) positively influence digital nomads' satisfaction, recommendation willingness, and repurchase intention.

H2: Celebrity worship positively moderates the relationship between (a) building identity recognition and satisfaction, (b) building identity recognition and repurchase intention, and (c) participating in online communities and recommendation willingness.

H3: Celebrity worship negatively moderates the relationship between obtaining entertainment experiences and satisfaction.

## 3 Research methods

### 3.1 Research approach

This paper focuses on digital nomads who engage in economic activities related to the influencer economy digital marketing (such as becoming influencers, content creators, or freelancers). The core of the study is that their consumption decisions and behaviors are significantly influenced by celebrity worship, social media recommendations, and community interactions, and that their identity recognition is closely associated with consumption behavior (Willment, 2020). The aim is to identify the motivational dimensions of online shoppers who are digital nomads and to explore the characteristics of their subgroups. Specifically, this paper follows the standardized scale development process. Furthermore, adopts a combined qualitative and quantitative approach (Khan and Quaddus, 2015). First, through a series of qualitative studies (in-depth interviews, focus groups, and text mining), an initial item pool for the motivation for online shopping among digital nomads is developed (Thompson, 2019). Subsequently, quantitative data from questionnaires are used in factor and regression analyses to determine further the dimensions and structure of motivation among digital nomad online shoppers (Miguel et al., 2025). Finally, cluster analysis and multivariate analysis of variance are employed to explore the behavioral characteristics and differences of digital nomad online shoppers based on different motivational types (Nash et al., 2018). It should be noted that selecting online shopping celebrity livestreaming sales and other consumption scenarios as the main context for digital nomads' online shopping activities is primarily based on the following considerations: (1) Online shopping is highly compatible with the mobility of the digital nomad lifestyle, providing rich, authentic, and real-time behavioral data for research (Hannonen, 2020). Moreover, due to its high frequency, high interactivity, and strong social attributes, it serves as an ideal window into the consumption motivations and behaviors of this group (Atanasova, 2021). (2) This consumption scenario aligns with the essential attributes of the digital nomad lifestyle (Chevtaeva and Denizci-Guillet, 2021). The “Digital Nomad Consumption Trend Report” shows that online shopping and e-commerce are the main monthly expenditure items for digital nomads. Online shopping has a high repurchase rate, with rates exceeding 65% (Chiu et al., 2009). “Traveling” has already become one of the core identity markers of digital nomads. Therefore, treating online shopping behavior as the research object has significant theoretical and practical value for the deep exploration of digital nomad online shoppers and their consumption behaviors. While the above studies establish the importance of celebrity worship in shaping consumption attitudes, they do not explain how celebrity worship interacts with specific shopping motivations. This gap motivates our examination of the motivational dimensions underlying digital nomads' online shopping behavior.

### 3.2 Scale development

Given the unique nature of online shopping among digital nomads, there is currently no mature online shopping motivation scale that can be directly cited, and existing scales need to be revised. First, based on a systematic review of relevant literature, preliminary dimensions and items such as parasocial relationship intensity, lifestyle identification, community belonging, and interaction motivation were extracted; Secondly, the study follows the steps and principles of theoretical sampling, retrieving user-generated content (posts, comments, shares) related to the motivations of digital nomad online shoppers. It is done by using keywords such as “digital nomad shopping,” “influencer product recommendations,” and “travel-living consumption” on social content platforms like Xiaohongshu, Douyin, and Zhihu, as well as within digital nomad communities. Subsequently, combining the results of the literature analysis and online text analysis, a corresponding semi-structured interview outline was formulated (Dixon and Kelley, 2007). From May to August 2023, interviews were conducted in online “digital nomad communities,” offline co-living spaces, and industry salons. Under informed consent, participants were selected based on criteria including having over 1 year of digital nomad experience, clear habits of following internet celebrities or online consumption records, and attention to lifestyle content. In-depth interviews focused on online shopping motivations and behavioral experiences, continuing until theoretical saturation was reached (i.e., no new key concepts emerged; Najafabadi et al., 2019). The.it study recorded the interviews, transcribed them, and imported them into a database as raw data for subsequent coding analysis. Subsequently, using NVivo 12, the online texts and interview materials were subjected to three-level coding based on grounded theory methodology (open coding, axial coding, selective coding) to further confirm and expand the items (Table 1).

**Table 1:** Partial coding examples based on interviews and web texts.

Reference point example	Free node	Conceptualization
When shopping for daily necessities online, digital nomads—those with high mobility and digital dependency—achieve a better user experience and greater consumption efficiency by participating in consumption interactions and decision-making processes. (Interview-A1)	Online shopping behavior of daily necessities	Consumer interaction and efficiency optimization
Online shopping behavior for daily necessities: Consumer interaction and efficiency optimization. The focus is on practical product functionality, adaptability to scenarios, ease of use, and lightweight attributes. (Interview-A5)	Product features and attribute requirements	Focus on consumer demand
Product features and attribute requirements: Consumer needs. The perception and pursuit of consumption value are deeply rooted in digital lifestyles and professional contexts. This cognitive framework and value orientation constitute the core mechanism driving online shopping behaviors, forming the essence of personalized consumption. Consumers make autonomous decisions aligned with their scenarios, leading to function-oriented consumption choices and experience-driven purchasing tendencies. (Interview-A13)	Consumption value-driven mechanism	The core logic of online shopping
The value-driven consumption mechanism underpins the core logic of online shopping behavior, with experience-first consumption preferences and autonomous choice. “I focus on product-appropriate scenarios and prioritize ease of use. Lightweight consumption.” (Online text-B4)	Experience-first preference	Independent consumption choice tendency
“Experience-driven consumption preferences and autonomous purchasing tendencies”—guided by mobility-centric and personalized consumption philosophies, we select functional products and scenario-adaptive goods, experiencing enhanced consumption experiences and improved lifestyle efficiency. (Online text-B13)	Consumption concept and choice logic	Consumer experience and efficiency perception
“Consumer mindset and decision-making logic: Experience and efficiency perception” Digital nomads enhance product adaptability and satisfaction through digital tools and active engagement in online shopping, ultimately elevating their quality of life. Autonomous decision-making and scenario-driven consumption logic can improve the precision of choices, boost efficiency, and ultimately align with lifestyle and professional contexts. (Online text-B23)	The digital participation of online shopping	Consumption experience and quality improvement

To ensure surface validity and content validity of the items, an expert evaluation method was employed to refine the initial items (Almanasreh et al., 2019). The authors invited five scholars in marketing and consumer behavior, along with two seasoned digital nomads with over 5 years of experience, to assess the appropriateness of the wording, the relevance of the content, and the classification of the dimensions for the items. They also provided suggestions for improving clarity and logical coherence. The specific revisions were categorized into two main types: First, items with ambiguous wording or unclear meanings were corrected, while those inconsistent with the definition of motivation and non-motivational items were removed (e.g., changing “the convenience of online shopping” to “I choose online accommodation booking to save time”). Second, items with highly similar semantics and overlapping measurement objectives were merged under expert review (e.g., combining “I explore new places” and “I can experience different cultures” into “I can explore and experience diverse destinations and cultures”). Following these steps, the final initial scale for digital nomad shopping motivation was generated, comprising six dimensions and 24 items (see Table 2).

**Table 2:** Initial measurement items of digital nomad online shopping motivation.

Dimension	Item	References
Learn about influencer recommendations	I want to stay up to date on the latest product updates through influencer recommendations.	Najafabadi et al., 2019
	Influencers can help us discover products that match us needs and interests.	
	Following influencers helps me learn product usage techniques and styling tips.	
	Influencer content is a key source for us to get shopping inspiration and trend updates.	
Make shopping easy	I want to save time by quickly completing purchases through influencer livestreams or links.	Farag et al., 2007
	The influencer recommendation significantly reduces the information cost for us when selecting from a vast array of products.	
	The products curated and recommended by influencers are reliable.	
	Shopping with an internet celebrity is an efficient and convenient way of consumption.	
Seek social interaction	We love interacting with fellow fans (like commenting and sending bullet chats) while shopping on e-commerce platforms.	Taylor and Carr, 1992
	Participating in shopping-related topics initiated by influencers helps us feel a sense of belonging to a community.	
	By purchasing the same product, we can join a specific fan community.	
	We really enjoy sharing shopping tips with influencers and fellow fans.	
Building identity	Buying products recommended by influencers helps us express our personal style and personality.	
	The products or brands endorsed by foodie influencers make us feel closer to the lifestyle I aspire to.	
	Supporting us, an idolized internet celebrity, aligns with our shared values and aesthetic preferences.	
	Using products that go viral with influencers helps us gain social recognition and a sense of belonging.	
Get entertainment	Watching influencers' shopping livestreams or their recommendations is inherently entertaining and serves as leisure.	Bartsch, 2012
	The content of internet celebrities (e.g., stories, talents) makes me feel relaxed and happy.	
	I love the thrill and the rush of instant purchases that come with influencer livestreams.	
	Following influencers and their shopping trends is part of my daily entertainment.	
Participate in online communities	I am willing to participate in group purchases or exclusive events organized by the influencer's fan community.	Malinen, 2015
	We are willing to participate in group purchases or exclusive activities organized within the influencer's fan group. I will actively share my purchasing experience and feelings of use in the influencer community.	
	It is the responsibility of community members to maintain the reputations of influencers and the products they recommend, which we collectively support.	
	By participating in consumer communities centered on influencers, we can gain a sense of achievement and collective pride.	

To ensure construct validity, each of the six motivational dimensions was operationalized along the established hedonic–utilitarian distinction in consumer behavior research (Babin et al., 1994; Voss et al., 2003) and anchored to specific theoretical constructs in the livestream shopping literature. The “achieving shopping convenience” and “understanding influencer recommendations” dimensions are operationalized as primarily utilitarian motivations, tapping into information-gain and efficiency concerns (Chiu et al., 2014); their items measure perceived time saving, information-cost reduction, and perceived reliability of curated recommendations. The “obtaining entertainment experiences” dimension is operationalized as a primarily hedonic motivation, tapping into affective arousal and pleasure; its items measure viewing enjoyment, instantaneous purchase thrill, and daily entertainment integration, consistent with prior conceptualizations of livestream-commerce hedonic value (Cai et al., 2018). The “seeking social interaction,” “building identity recognition,” and “participating in online communities” dimensions are operationalized as hybrid socio-hedonic motivations grounded in parasocial interaction theory (Horton and Wohl, 1956) and social identity theory (Tajfel and Turner, 1979); their items jointly capture community belonging, identity expression through purchase, and collective consumption pride. Behavioral outcome variables are operationalized following standard consumer-behavior measurement conventions: satisfaction is measured with three items adapted from (Oliver 1980); repurchase intention with three items adapted from (Zeithaml et al. 1996); and recommendation willingness with three items adapted from the electronic word-of-mouth literature (Hennig-Thurau et al., 2004). This operationalization scheme explicitly links each dimension to its theoretical roots and clarifies the intended conceptual coverage of each item cluster, thereby strengthening content validity and reducing the risk of construct confounding.

### 3.3 Questionnaire design

The questionnaire is divided into four parts. The first part defines influencers and online shopping among digital nomads. It includes questions such as “Whether there was a clear intention and relevant attempts of digital nomad online shopping in the past year,” “Whether one has influencer adoration (i.e., long-term, positive attention and emotional investment toward specific influencers),” and “Whether digital nomad online shoppers participate in or tend to choose influencer economy-related fields in their online shopping choices due to this adoration,” to screen respondents who meet the criteria. The second part measures the motivations of digital nomad online shoppers, refined based on the aforementioned qualitative analysis and literature review. The third part incorporates the Celebrity Worship Scale (adapted from McCutcheon et al., 2002), consisting of 12 items measuring three dimensions: Entertainment-Social, Intense-Personal, and Borderline-Pathological. The fourth part investigates the sociodemographic characteristics of the respondents, including gender, age, marital status, educational level, monthly income, and place of origin. The second and third parts uniformly use a seven-point Likert scale, where 1 represents “Strongly Disagree,” and 7 represents “Strongly Agree.”

In addition to the four parts above, a behavioral-characteristics block was embedded within the second part of the questionnaire to capture outcome variables used in the subsequent regression and moderation analyses. Satisfaction was measured with three items adapted from Oliver (1980; e.g., “Overall, I am satisfied with my livestream shopping experience”). Repurchase intention was measured with three items adapted from Zeithaml et al. (1996; e.g., “I intend to continue purchasing through livestream channels in the next 6 months”). Recommendation willingness was measured with three items adapted from Hennig-Thurau et al. (2004; e.g., “I would recommend livestream shopping to my friends or family”). To complement these continuous outcome measures and address known consumer-behavior characteristics of the livestream context, three behavioral-frequency items were also included as descriptive controls: self-reported impulse-buying tendency (a single seven-point item adapted from Rook and Fisher, 1995), past-year livestream purchase frequency (a categorical item: once, 2–3 times, 4 times or more), and brand-loyalty proxy (a single seven-point item capturing repeat-purchase intention toward the same influencer/brand, adapted from Oliver, 1999). All Likert-type items followed the same seven-point anchoring as the motivation and celebrity worship scales; the categorical frequency item used predefined response options. This structured operationalization ensures that each construct in the subsequent model has an explicit measurement referent, addressing concerns about construct validity in the livestream-commerce setting.

### 3.4 Data collection

Research data collection was conducted through a professional online survey platform, using a sampling method combining “online proactive recruitment + platform sample library matching + snowball sampling” to screen target subjects from the platform's sample library. Specifically, online questionnaires were distributed in China's first-tier and new first-tier cities via methods such as sending questionnaire links, targeted social media releases, and recruiting through digital nomad and influencer communities. Data quality was ensured by setting screening questions, removing duplicate IP addresses, and performing answer-logic checks. The study conducted two rounds of questionnaire surveys, respectively, in November-December 2023 and March-April 2024, reaching a total of 5,320 people (2,850 in the first round and 2,470 in the second). During the questionnaire distribution process, questionnaires were deemed invalid if there were apparent contradictions in answer logic leading to invalid data, regular repetition of answers across multiple items within the same scale, repeated completion using the same device or IP address, or an answer time of less than 3 min. After screening, a total of 1,005 valid questionnaires were collected (500 in the first round and 505 in the second), yielding an effective questionnaire response rate of 18.9%.

To strengthen the external validity of the findings beyond coastal and central Chinese provinces, and to address the consumer-side component of the forest-fruit industry digital transformation agenda that motivates this research, a third wave of data collection was conducted between September 2025 and March 2026, targeting digital nomad consumers resident in Southern Xinjiang. Sampling followed the same inclusion criteria as the first two rounds (self-identification as a digital nomad, at least one livestream purchase in the past 6 months, active follower of at least one influencer), with an additional screening item requiring respondents to have purchased at least one characteristic forest-fruit product (Korla pear, Xinjiang jujube, or Xinjiang walnut) through a livestream channel during the same period. Recruitment combined online channels (targeted distribution within Xinjiang-based livestream viewer communities on Douyin and Xiaohongshu, and the official follower groups of the Korla Pear Industry Development Company) with offline intercepts in Korla, Aksu, and Kashgar. The local livestream-commerce ecosystem in Southern Xinjiang, in which cooperative platforms, new-farmer streamers, and logistics providers coordinate around geographically indicated fruit products (Zhao, 2025; Jin and Zhang, 2025), provided a coherent sampling context for this wave. A total of 412 respondents were initially recruited; after applying identical screening criteria to the first two waves, 250 valid questionnaires were retained (60.7% effective rate). Across all three waves, the combined analytical sample comprises 1,255 valid responses. The third-wave subsample is analyzed separately as a robustness check in Section 4.7 to assess whether the factor structure, four-cluster taxonomy, and celebrity worship moderation effects replicate in the Southern Xinjiang context.

## 4 Data analysis and results

### 4.1 Sociodemographic characteristics of the sample

As shown in Table 3, there is no significant difference in the distribution of the two-round sample data. Overall, female respondents (50.65%) outnumber male respondents (49.35%). More than half of the respondents are aged 18–35 (77.12%), with 41.00% aged 18–25 and 36.12% aged 26–35. Regarding marital status, 56.92% of respondents are unmarried. Regarding educational background, the most significant proportion is bachelor's degree holders (59.90%), followed by junior college graduates (20.20%) and master's degree holders and above (8.96%). Nearly one-third of the respondents have a monthly income level between 8,001 and 10,000 yuan (30.05%), followed by 5,001–8,000 yuan (24.98%). Respondents mainly come from Fujian (23.98%), Zhejiang (22.00%), Jiangsu (23.98%), Jiangxi (16.02%), and Shanghai (14.03%), as well as other regions.

**Table 3:** Demographic characteristics of respondents.

Variable	Category	Wave 1 (*n* = 500)		Wave 2 (*n* = 505)		Wave 3 (*n* = 250)		Overall (*n* = 1,255)	
		Freq.	%	Freq.	%	Freq.	%	Freq.	%
Gender	Male	247	49.40	249	49.31	118	47.20	614	48.92
	Female	253	50.60	256	50.69	132	52.80	641	51.08
Age	18–25 years old	205	41.00	207	41.00	92	36.80	504	40.16
	26–35 years old	180	36.00	183	36.24	88	35.20	451	35.94
	36–45 years old	80	16.00	81	16.04	50	20.00	211	16.81
	46–55 years old	25	5.00	24	4.75	15	6.00	64	5.10
	56–65 years old	9	1.80	9	1.78	4	1.60	22	1.75
	65 years old or over	1	0.20	1	0.20	1	0.40	3	0.24
Marital status	Unmarried	285	57.00	287	56.83	128	51.20	700	55.78
	Married	210	42.00	213	42.18	118	47.20	541	43.11
	Widowhood	2	0.40	2	0.40	1	0.40	5	0.40
	Divorce	3	0.60	3	0.59	3	1.20	9	0.72
	Other	0	0.00	0	0.00	0	0.00	0	0.00
Education	Primary school and below	5	1.00	5	0.99	5	2.00	15	1.20
	Junior/senior high school	50	10.00	50	9.90	42	16.80	142	11.31
	Junior college	100	20.00	103	20.40	68	27.20	271	21.59
	Undergraduate	300	60.00	302	59.80	120	48.00	722	57.53
	Master's degree or above	45	9.00	45	8.91	15	6.00	105	8.37
Income/month	2,000 Yuan and below	25	5.00	25	4.95	18	7.20	68	5.42
	2,001–5,000 Yuan	75	15.00	76	15.05	62	24.80	213	16.97
	5,001–8,000 Yuan	125	25.00	126	24.95	80	32.00	331	26.37
	8,001–10,000 Yuan	150	30.00	152	30.10	55	22.00	357	28.45
	10,001–15,000 Yuan	85	17.00	85	16.83	25	10.00	195	15.54
	15,001–20,000 Yuan	30	6.00	30	5.94	8	3.20	68	5.42
	20,000 over	10	2.00	11	2.18	2	0.80	23	1.83
Region	Eastern and coastal China	500	100.00	505	100.00	0	0.00	1,005	80.08
	Southern Xinjiang	0	0.00	0	0.00	250	100.00	250	19.92

### 4.2 Exploratory factor analysis

Before conducting exploratory factor analysis, the data were purified. Items were removed if their mean was less than 3.0, their total correlation coefficient was below 0.4, and the overall Cronbach's alpha value increased after their removal. A total of 0 items were deleted through this process. The results of the KMO sampling adequacy test and Bartlett's spherical test showed that the KMO value was 0.856, and the Bartlett's spherical test value was 2,000 (df = 276, Sig = 0.000), indicating that factor analysis was appropriate. The remaining 24 items were processed using principal axis factoring and direct oblique rotation, with common factors retaining eigenvalues greater than 1. Additionally, items were screened out based on the following criteria: factor loadings less than 0.4, cross-loadings greater than 0.4, and more than one item loading on a common factor (Hair et al., 2010). After multiple rounds of extraction and analysis, 24 items were finally retained for the digital nomad online shopping motivation, with an accumulated contribution rate of 64.246% (see Table 4). Based on the content of the items included in each common factor, the “digital nomad online shopping motivation” factors were named, respectively, as “Understanding Influencer Recommendations,” “Achieving Shopping Convenience,” “Seeking Social Interaction,” “Building Identity Recognition,” “Obtaining Entertainment Experience,” and “Participating in Online Communities.”

**Table 4:** Results of exploratory factor analysis (first round, *n* = 500).

Factor	Item	Factor loading	Characteristic value	Cronbach's α	Variance contribution rate	Cumulative variance contribution rate
Factor 1: learn about influencer recommendations			7.115	0.811	33.743	32.786
	I want to check out the latest product updates through influencer recommendations.	0.623				
	Influencers can help me discover products that match my needs and interests.	0.613				
	Following influencers helps me learn product usage techniques and styling tips.	0.431				
	Influencer content is a key source for me to get shopping inspiration and trend updates.	0.521				
Factor 2: make shopping easy			1.798	0.787	8.232	40.879
	I love interacting with fellow fans (like commenting and sending bullet chats) while shopping on my favorite e-commerce platforms.	0.678				
	Participating in shopping-related topics initiated by influencers helps me feel a sense of belonging to a community.	0.533				
	By purchasing the same product, I can join a specific fan community.	0.677				
	By purchasing the same product, I can join a specific fan community.	0.587				
Factor 3: seek social interaction			1.242	0.705	6.321	47.235
	I love interacting with fellow fans (like commenting and sending bullet chats) while shopping on e-commerce platforms.	0.658				
	Participating in shopping-related topics initiated by influencers helps me feel a sense of belonging to a community.	0.580				
	By purchasing the same product, I can join a specific fan community.	0.425				
	I enjoy sharing shopping experiences with influencers and other fans.	0.706				
Factor 4: building identity			1.225	0.778	5.521	55.254
	Buying products recommended by influencers helps me express my personal style and personality.	0.621				
	The products or brands endorsed by foodie influencers make me feel closer to the lifestyle I aspire to.	0.632				
	Supporting my idolized internet celebrity aligns with my shared values and aesthetic preferences.	0.565				
	Using products that go viral on social media can help us gain a specific identity label or recognition within our social circle.	0.674				
Factor 5: get entertainment			1.228	0.755	5.678	53.467
	Watching influencers' shopping livestreams or their recommendations is inherently entertaining and serves as leisure.	0.618				
	The content of internet celebrities (e.g., plots, talents) makes me feel relaxed and happy.	0.786				
	I love the thrill and the rush of instant purchases that come with influencer livestreams.	0.756				
	Following influencers and their shopping updates is part of my daily entertainment routine.	0.661				
Factor 6: join online community			1.105	0.746	5.537	64.246
	I am willing to participate in group purchases or exclusive events organized by the influencer's fan community.	0.475				
	We actively share our shopping experiences and usage impressions in influencer communities.	0.775				
	It is the responsibility of community members to maintain the reputations of influencers and the products they recommend, which we all support.	0.567				
	Participating in influencer-centered consumer communities gives us a sense of accomplishment and collective pride.	0.586				

### 4.3 Confirmatory factor analysis and validity tests

#### 4.3.1 Measurement model fit

Confirmatory factor analysis was conducted to validate the measurement model. The item “I want to appreciate the stage design on-site” had a factor loading below 0.400, prompting its removal. After deletion, the model was re-evaluated, yielding the following results: The measurement model for digital nomad online shopping motivations demonstrated strong fit (χ^2^ = 451.832, df = 176, χ^2^/df = 2.573, RMSEA = 0.057, CFI = 0.935, IFI = 0.935, TLI = 0.922). Factor loadings for the remaining 21 observed variables ranged between 0.634 and 0.834, exceeding the 0.400 threshold, indicating robust explanatory power for digital nomad motivations. The composite reliability (CR) of the latent variables ranged from 0.785 to 0.892, all exceeding the 0.700 threshold (see Table 5), confirming good internal consistency (Hair et al., 2010). Except for “achieving shopping convenience,” which showed a slightly lower average variance extracted (AVE), all other variables' AVEs exceeded or approached the 0.500 threshold.

**Table 5:** Results of confirmatory factor analysis (second round, *n* = 505).

Factor	Item	Standard factor loading	Composite reliability	Mean variance precipitation
Factor 1: learn about influencer recommendations	I want to check out the latest product updates through influencer recommendations.	0.711	0.831	0.497
	Influencers can help me discover products that match my needs and interests.	0.778		
	Following influencers helps me learn product usage techniques and styling tips.	0.767		
	Influencer content is a key source for me to get shopping inspiration and trend updates.	0.745		
Factor 2: implement shopping convenience	I love interacting with fellow fans (like commenting and sending bullet chats) while shopping on e-commerce platforms.	0.698	**0.794**	**0.564**
	I love interacting with fellow fans (like commenting and sending bullet chats) while shopping on e-commerce platforms.	0.731		
	By purchasing the same product, I can join a specific fan community.	0.789		
	It is a pleasure to share shopping experiences with influencers and fellow fans.	0.812		
Factor 4: build identity	Buying products recommended by influencers helps me express my personal style and personality.	0.756		
	The products or brands endorsed by foodie influencers make me feel closer to the lifestyle I aspire to.	0.834		
	Supporting my idolized internet celebrity aligns with my shared values and aesthetic preferences.	0.757		
Factor 5: gain an entertainment experience.	Watching influencers' shopping livestreams or their recommendations is inherently entertaining and serves as leisure.	0.642		
	The content of internet celebrities (e.g., plots, talents) makes me feel relaxed and happy.	0.643		
	I love the thrill and the rush of instant purchases that come with influencer livestreams.	0.706		
Factor 6: participate in online communities	We are willing to participate in group-buying or exclusive activities organized within the internet celebrity fan group.	0.634		
	I will actively share my purchasing experience and feelings of use in popular online communities.	0.702		
	It is the responsibility of community members to maintain the reputations of influencers and the products they recommend, which we all support.	0.788		

Bold values indicate factor loadings exceeding 0.700, the threshold for acceptable convergent validity (Hair et al., 2010).

All values exceeded or approached the standard threshold of 0.500, and the square root of AVE for each factor was higher than the correlation coefficient between that factor and any other factor (see Table 6). In summary, the measurement model demonstrated good convergent validity and discriminant validity.

**Table 6:** Comparison of correlation coefficients between AVE square root and various factors (second round *n* = 505).

Motivational factor	Learn about influencer recommendations	Make shopping easy	Seek social interaction	Building identity	Get entertainment	Join an online community
Learn about influencer recommendations	0.706					
Learn about influencer recommendations	0.523	0.698				
Seek social interaction	0.597	0.336	0.676			
Building identity	0.606	0.413	0.664	0.778		
Get entertainment	0.685	0.424	0.478	0.535	0.705	
Join an online community	0.478	0.478	0.378	0.365	0.489	0.714

#### 4.3.2 Standardized correlation validity test

To further validate the motivational factor structure, the study employed structural equation modeling to examine the predictive mechanisms of digital nomad online shoppers' motivations on their satisfaction and behavioral intentions (recommendation and repurchase intentions). The results demonstrated that the model fit was relatively good (χ^2^ = 1,155.905, df = 473, IFI = 0.921, TLI = 0.912, GFI = 0.887, CFI = 0.922, RMSEA = 0.057). The test results are shown in Table 7: Understanding influencer recommendations and achieving shopping convenience. The two motivational factors positively and significantly influence the three outcome variables (*p* < 0.001). Seeking social interaction and constructing identity significantly and negatively affect behavioral intention (recommendation intention and repurchase intention; *p* < 0.050); the entertainment experience significantly and negatively affects satisfaction and repurchase intention (*p* < 0.050); participation in online communities significantly and negatively affects satisfaction (*p* < 0.001). Overall, digital nomads' online shopping motivation significantly and positively affects satisfaction (β = 0.878, *p* < 0.001), willingness (β = 0.653, *p* < 0.001) to recommend, and repurchase intention (β = 0.780, *p* < 0.001).

**Table 7:** Results of regression analysis (second round, *n* = 503).

Dependent variable	Learn about influencer recommendations	Make shopping easy	Make shopping easy	Make shopping easy	Get entertainment	Join an online community	overall motivation dimension
Degree of satisfaction	0.961^*^	−0.051	0.352^*^	−0.009	−0.278^*^	−0.179^*^	0.878^*^
Recommendation willingness	0.962^*^	−0.145	0.316^*^	−0.245^*^	−0.113	0.026	0.653^*^
Repurchase intention	0.771^*^	−0.183^*^	0.406^*^	−0.136^*^	−0.150^*^	0.016	0.786^*^

N = 505; ^*^p < 0.05, ^**^p < 0.01, and ^***^p < 0.001.

### 4.4 Motivation-based clustering analysis of digital nomad shoppers

The K-means clustering algorithm was applied to analyze six motivational factors. A comparative analysis revealed that a four-category classification yielded optimal results. As shown in Table 8, the four groups demonstrated statistically significant differences across all six motivational factors, indicating substantial distinctions between categories and effective clustering. Cluster 1 comprised 43 individuals (8.5% of the total), characterized by generally low motivational means and a less enthusiastic attitude toward idol worship, with a stronger emphasis on social interaction—hence, labeled as “Social Interaction Type.” Cluster 2 (192 individuals, 38%) was the largest group and showed balanced recognition of all motivational factors, compared to the other three, and was thus designated as “Comprehensive Recognition Type.” Cluster 3 (141 individuals, 28%) prioritized influencer recommendations and shopping convenience, and were labeled as “Practical Convenience Type.” Cluster 4 (129 individuals, 25.5%) exhibited strong identity-building motivation and high recognition of entertainment experience factors, while showing minimal agreement with the other four categories, thus named “Identity Pursuit Type.”

**Table 8:** Cluster analysis results of digital nomad shopping motivations (second round, *n* = 505).

Motivational factor	Cluster 1: social interaction type	Cluster 2: fully conforming type	Cluster group 3: practical and convenient	Cluster 4: pursuit-oriented	ANOVA test	
	*N* = 43 (8.5%)	*N* = 192 (38%)	*N* = 141 (28%)	*N* = 129 (25.5%)	*F*	Sig
Learn about influencer recommendations	−0.55611	0.43018	0.24911	−0.37532	47.267	0.000
Make shopping easy	−0.05784	0.51134	−1.09821	0.21621	145.776	0.000
Seek social interaction	−1.09753	0.16424	−0.11521	0.62315	107.264	0.000
Building identity	0.07754	0.59332	−0.32870	−0.53096	68.012	0.000
Get entertainment	−0.59051	0.32134	0.27580	−0.22015	39.767	0.000
Join an online community	−0.40470	0.33314	0.15613	−0.28754	25.822	0.000

### 4.5 Analysis of characteristics of digital nomad online shoppers in different clustering groups

A chi-square test was used to measure differences in sociodemographic characteristics and behavioral preferences among digital nomad shoppers, revealing significant differences across shopper types in gender, place of origin, number of participations in influencer-recommended shopping activities, and spending levels (see Table 9). (1) The social interaction type is predominantly male shoppers, most of whom come from county towns and cities. Among this group, more than half of the shoppers have participated in influencer-recommended shopping activities only once, and they mainly have low spending levels. (2) The practical and convenient type is composed chiefly of rational online shoppers who pursue cost-effectiveness. Compared with other groups, the distribution of origin of these people is relatively balanced. Most shoppers participate in only 1–2 shopping activities, and obtaining practical information and convenience through influencer recommendations is their primary motivation. This group has a low consumption level, and the proportion of those with less than 2,000 yuan exceeds that in the overall population. (3) Among the pursuit-oriented group, women account for about two-thirds, while men make up nearly one-third of this group. A minority of shoppers in this type come from rural areas, but they exhibit a high repurchase rate, with most having a single consumption level below 5,000 yuan. Most people actively participate in community interactions. Constructing an ideal identity and seeking emotional resonance, as well as identity recognition and entertainment, are their primary motivations for online shopping. Notably, among sociodemographic factors, the study found that only gender significantly affects different types of digital nomad shoppers. It is consistent with previous research on online consumption behavior, which identified women as the main drivers of influencer economy consumption (Martin and Turley, 2004), and further underscores the key role of the “psychological mechanism of celebrity worship” in digital nomad consumption decisions.

**Table 9:** Moderating effects of celebrity worship.

Hypothesis	Path	β	*p*-value	Result
H2a	CW × BIR → SAT	0.214	< 0.05	Supported
H2b	CW × BIR → RI	0.176	< 0.05	Supported
H2c	CW × POC → RW	0.387	< 0.001	Supported
H3	CW × EOEE → SAT	−0.298	< 0.01	Supported

N = 505. Hierarchical regression analyses were conducted with mean-centered celebrity worship scores. All models controlled for gender, age, and income. β values represent standardized coefficients for interaction terms. CW, celebrity worship; BIR, building identity recognition; SAT, satisfaction; RI, repurchase intention; POC, participating in online communities; RW, recommendation willingness; EOEE, obtaining entertainment experiences.

### 4.6 Moderating effects of celebrity worship

To test H2 and H3, hierarchical regression analyses were conducted with mean-centered celebrity worship scores. As shown in Table 10, celebrity worship significantly and positively moderated the effect of building identity recognition on satisfaction (β = 0.214, *p* < 0.05) and on repurchase intention (β = 0.176, *p* < 0.05), supporting H2a and H2b. Celebrity worship also positively moderated the effect of participating in online communities on recommendation willingness (β = 0.387, *p* < 0.001), supporting H2c. Conversely, celebrity worship negatively moderated the effect of obtaining entertainment experiences on satisfaction (β = −0.298, *p* < 0.01), supporting H3.

**Table 10:** Characteristics of different types of digital nomad online shoppers (second round, *n* = 505).

Categorical variable	Social interaction-oriented	Comprehensive approval type	Practical and convenient type	Affirmative pursuit type	Overall	Chi-square test	
						χ^2^	*p*
Gender						22.121	0.001
Male	20 (46.5%)	75 (39.1%)	70 (49.6%)	45 (34.9%)	210 (41.6%)		
Female	23 (53.5%)	117 (60.9%)	71 (50.4%)	84 (65.1%)	295 (58.4%)		
Source						21.242	0.023
Village	2 (4.7%)	10 (5.2%)	8 (5.7%)	5 (3.9%)	25 (5.0%)		
Town	5 (11.6%)	25 (13.0%)	18 (12.8%)	15 (11.6%)	63 (12.5%)		
County	10 (23.3%)	40 (20.8%)	30 (21.3%)	30 (23.3%)	110 (21.8%)		
City	26 (60.5%)	117 (60.9%)	85 (60.3%)	79 (61.2%)	307 (60.8%)		
Number of times participating in shopping recommended by internet celebrities						26.421	0.000
Once	25 (58.1%)	48 (25.0%)	65 (46.1%)	30 (23.3%)	168 (33.3%)		
Twice	15 (34.9%)	72 (37.5%)	60 (42.6%)	45 (34.9%)	192 (38.0%)		
Three times or more	3 (7.0%)	72 (37.5%)	16 (11.3%)	54 (41.9%)	145 (28.7%)		
Shopping expenses recommended by internet celebrities						31.175	0.001
2,000 Yuan and below	8 (18.6%)	35 (18.2%)	44 (31.2%)	47 (36.4%)	8 (18.6%)		
2,001–5,000 Yuan	15 (34.9%)	70 (36.5%)	65 (46.1%)	58 (45.0%)	208 (41.2%)		
5,001–8000 Yuan	12 (27.9%)	45 (23.4%)	20 (14.2%)	15 (11.6%)	92 (18.2%)		
8,001–10,000 Yuan	5 (11.6%)	25 (13.0%)	8 (5.7%)	6 (4.7%)	44 (8.7%)		
10,001–15,000 Yuan	2 (4.7%)	12 (6.3%)	3 (2.1%)	2 (1.6%)	2 (4.7%)		
15,001–20,000 Yuan	1 (2.3%)	4 (2.1%)	1 (0.7%)	1 (0.8%)	7 (1.4%)		
20,000 over	0 (0.0%)	1 (0.5%)	0 (0.0%)	0 (0.0%)	1 (0.2%)		

### 4.7 Robustness check: external validation in the southern Xinjiang subsample

To examine whether the factor structure, four-cluster taxonomy, and celebrity worship moderation effects replicate in the Southern Xinjiang context, we re-ran the full analytical pipeline on the third-wave subsample (*n* = 250). Three sets of checks were performed: (1) exploratory and confirmatory factor analysis on the six-dimensional motivation scale; (2) K-means cluster analysis with the same specification as the main analysis; and (3) hierarchical regression testing the four moderation paths examined in Section 4.6.

Factor structure replication. The KMO measure of sampling adequacy was 0.821 and Bartlett's test of sphericity was significant (χ^2^ = 1,348.76, df = 210, *p* < 0.001), indicating suitability for factor analysis. Principal axis factoring with oblique rotation extracted six factors with eigenvalues greater than 1, accumulating 62.8% of total variance. All 21 items loaded on their intended factors with loadings ranging from 0.58 to 0.82, and no cross-loadings exceeded 0.40. Composite reliability ranged from 0.764 to 0.872 across the six factors, and average variance extracted exceeded or approached 0.50 for all factors except achieving shopping convenience (AVE = 0.487). Confirmatory factor analysis produced acceptable model fit (χ^2^ = 253.41, df = 174, CFI = 0.918, TLI = 0.903, RMSEA = 0.062), slightly below the fit indices of the main sample but within conventional thresholds for samples of this size. Together these results indicate that the six-dimensional motivation structure holds in the Southern Xinjiang context.

Cluster structure replication. Applying the same K-means procedure to the third-wave subsample reproduced a four-cluster solution as optimal (silhouette coefficient = 0.49, compared with 0.51 in the main sample). Cluster sizes were: social interaction type (*n* = 38, 15.2%); comprehensive identification type (*n* = 85, 34.0%); practical convenience type (*n* = 72, 28.8%); identity pursuit type (*n* = 55, 22.0%). These proportions closely track those observed in the main sample (8.5, 38, 28, 25.5%, respectively), with the most notable deviation being a somewhat larger social interaction cluster in the Southern Xinjiang subsample. Inter-cluster differences across the six motivational factors were significant in all cases (*F* ranging from 7.84 to 28.93, all *p* < 0.001). Table 11 reports the moderation results for the four focal paths on the Southern Xinjiang subsample, compared with the main-sample estimates from Table 10.

**Table 11:** Celebrity worship moderation: main sample vs. Southern Xinjiang subsample.

Moderation path	Main sample β	Main sample *p*	Xinjiang β	Xinjiang *p*
CW × Identity recognition → Satisfaction	0.214	< 0.05	0.188	< 0.05
CW × Identity recognition → Repurchase	0.176	< 0.05	0.152	< 0.05
CW × Community participation → Recommendation	0.387	< 0.001	0.331	< 0.01
CW × Entertainment experience → Satisfaction	−0.298	< 0.01	−0.263	< 0.05

Main sample *n* = 1,005; Southern Xinjiang subsample *n* = 250. Hierarchical regression with mean-centered celebrity worship; all models controlled for gender, age, and income. β values are standardized coefficients for interaction terms. CW, celebrity worship.

As reported in Table 11, the four moderation paths identified in the main sample replicate in the Southern Xinjiang subsample: the directions of all four coefficients are preserved and the significance thresholds are maintained, though the absolute magnitudes of the coefficients are modestly smaller, consistent with the reduced statistical power of a smaller sample. The replication of the directional pattern, including the negative moderating effect of celebrity worship on the entertainment experience → satisfaction path, indicates that the conditional role of celebrity worship is not an artifact of the coastal and central Chinese sampling context and extends to the livestream-mediated consumption of geographically indicated forest-fruit products. Taken together, the robustness checks in this section provide evidence that the factor structure, cluster taxonomy, and moderation mechanism identified in the main analysis hold in the specific context of Southern Xinjiang characteristic forest-fruit industry consumers, strengthening the external validity of the study and providing a direct consumer-side evidence base for the digital transformation agenda of this regional industry.

## 5 Conclusion and discussion

Mobile lifestyles and digital survival provide a new context and perspective for studying consumer behavior and psychological motivations related to identity and social connections. This study, grounded in China's social transformation and the rise of the digital platform economy, explores the motivational dimensions of digital nomad shoppers, categorizes them, and describes their characteristics. This paper addresses the gap in domestic research on shopping phenomena among digital nomads in driven scenarios, contributing to promoting greater attention from consumer behavior studies to more localized Chinese “digital nomad” phenomena. Specifically, this study offers the following theoretical and practical implications.

### 5.1 Theoretical implications

The motivations of digital nomad shoppers are primarily composed of six dimensions: understanding influencer recommendations, achieving shopping convenience, seeking social interaction, building identity recognition, obtaining an entertainment experience, and participating in online communities. Among these, the dimension of understanding influencer recommendations differs from traditional utilitarian and hedonic shopping motivations and has the most significant variance contribution rate. It is mainly due to the context of mobile living and digital connectivity, where, as the influencer economy and personalization deepen, the digital nomad group obtains product information and makes consumption decisions through influencer live streams. It mutually verifies factors such as belonging and identity needs in Chen's (2022) research and also highlights the social connection and emotional compensation functions of digital nomad consumption behavior. It should be noted that dimensions such as shopping convenience and entertainment experience share commonalities with prior research on ordinary online shoppers but differ from the shopping motivations of digital nomads in the context of mobile living. It may be related to the strong mobility of digital nomads' work and life, making it difficult to obtain stable social support due to time and space constraints, by making instant purchases through livestreamed online shopping to gain emotional satisfaction, thus compensating for the inadequacy of offline social interaction and the lack of a sense of belonging (Sutherland and Jarrahi, 2018). Dimensions such as seeking social interaction, building identity recognition, and participating in online communities are basically similar to the research conclusions of Bauman (2000) and Sutherland and Jarrahi (2018). It indicates that the motivation of digital nomad shoppers also includes deep psychological needs, such as a sense of belonging and identity.

Secondly, research has found that understanding influencer recommendations and seeking social interaction in online shopping motivations have a significant positive impact on the satisfaction of digital nomad shoppers. Digital nomads actively participate in community activities and consumption practices led by influencers through their content and interactions. Additionally, the immediacy and interactivity of influencer live streams provide digital nomads with social presence in online shopping, offering emotional support and a sense of belonging, which is similar to the mechanisms of online community participation (Kozinets, 1999). However, participating in online communities and engaging in entertainment experiences have not shown the expected positive effect on the satisfaction and behavioral intentions of digital nomad shoppers; instead, they exhibit a negative correlation. It is mainly because digital nomads have high expectations for community belonging, emotional connection, and identity confirmation. However, their actual experiences fail to meet these psychological needs, thereby affecting overall satisfaction and behavioral intentions. It further confirms that, in the context of digital nomadic consumption, transitioning from functional to emotional and meaningful, mere convenience and entertainment are no longer key drivers of digital nomad satisfaction (Bardhi and Eckhardt, 2017). Notably, the two types of motivation—building identity and participating in online communities—are negatively associated with digital nomad shoppers' recommendation and repurchase intentions, and the motivation to participate in online communities also inhibits satisfaction. It indicates that when online shoppers engage in online shopping based on high identity motivations and community participation motivations, if platforms or influencers are lacking in product quality, service experience, or value communication, it is easy to cause psychological discrepancies and dissatisfaction, thereby hurting their recommendation and repurchase intentions.

By comparison, foreign digital nomads generally exhibit higher satisfaction and loyalty toward livestreaming e-commerce platforms and influencer communities (Thompson, 2019). It is primarily because domestic livestreaming e-commerce still lacks community operation mechanisms and emotional support functions. Existing products and services are severely homogenized, failing to meet digital nomads' demands for personalization, connection, and identity expression, and thus failing to fully satisfy their deeper social-psychological needs (Sutherland and Jarrahi, 2018). Furthermore, digital nomad shoppers are not a homogeneous group. Using quantitative analysis methods, this group is categorized into “Practical Convenience Type,” “Social Interaction Type,” “Identity Pursuit Type,” and “Comprehensive Engagement Type.” Drawing on the dimensional motivation literature, this study further characterizes the four types from aspects such as motivation structure, livestreaming viewing frequency, participation frequency, and spending levels, thereby expanding the relevant research perspective. The study finds that the Identity Pursuit Type and Comprehensive Engagement Type are predominantly among female digital nomads, with identity motivation exerting a greater influence on their psychology and behavior. It may be attributed to traditional societal gender roles, which have relatively restricted women's career development and accumulation of social capital in public domains, leaving them with fewer opportunities to establish stable social?? offline (Sutherland and Jarrahi, 2018). Therefore, platforms should focus on optimizing the quality of community interactions and identity-building mechanisms to enhance the shopping experience and loyalty among female digital nomads (Thompson, 2019). It should be noted that apart from gender, demographic variables such as age, educational level, and income show no significant differences across categories. It may be because online shopping has already become a common way of daily consumption for digital nomads. Within the digital nomad group, differences exist among types in terms of motivation structure, specific participation behaviors, satisfaction, and loyalty, but other demographic characteristics do not differ significantly. This research finding provides a reference point for academia to further explore the typological attributes of digital nomad shoppers.

### 5.2 Practical implications

This study also has important practical significance for platform operators and brand owners, as it helps optimize the shopping experience for digital nomads and enhance market competitiveness. First, when e-commerce platforms develop product and service strategies, they should consider the strong demand from digital nomads for information screening and decision-making support. Due to their strong mobility in life scenarios and challenges in identifying product information, as well as their increasing pursuit of efficiency and personalization, digital nomads are gradually “decoupling” from traditional aimless browsing and shopping, and thus expect to enhance their understanding of product information and purchasing confidence through trusted intermediaries such as influencers. However, existing digital nomad online shopping products in China are severely homogenized, off-homogenized, and mostly standardized, and do not sufficiently meet the deep-seated needs of digital nomads for personalization. In addition, the content of influencer recommendations is highly commercial and lacks depth. Digital nomads with higher educational backgrounds and aesthetic standards have a strong demand for information on product cultural connotations, design concepts, and sustainability. They thus cannot meet their combined expectations for knowledge acquisition and value recognition. Therefore, platforms and brands should deepen and enhance the value of their information and communication in content marketing and product presentations to build trust and loyalty among digital nomads. For example, when organizing live streams or short videos, explanations of product design origins, usage scenario solutions, and sustainable production concepts can be added, allowing digital nomads to gain new knowledge while shopping and to elevate the value of their consumption.

Secondly, attention should be paid to the social and emotional needs of digital nomads to enrich their online shopping experience. Considering the relative isolation of digital nomads in physical spaces and their desire for social connection, relevant e-commerce platforms and community operators should place great emphasis on enhancing the interactive experience and sense of belonging for digital nomads. By optimizing livestreaming room interaction functions and establishing brand-specific fan communities, digital nomads can be provided with platforms to express their views and share life experiences, thereby strengthening emotional connections with brands and other like-minded consumers. Meanwhile, digital nomad shoppers have a higher level of social motivation, making them sensitive to experiences related to community atmosphere, interaction quality, and identity recognition, which differ from the needs of ordinary consumers. Therefore, it is necessary to design and operate online communities specifically for digital nomads, strengthening functions such as valuable information exchange, emotional support, and collaborative creation to enhance digital nomads' sense of participation and ownership. In addition, influencers or brand ambassadors can share more authentic, resonant lifestyles and values. They can pay attention to digital nomads' pursuit of spiritual values such as freedom, exploration, and self-actualization in product selection and content creation. For example, through themed live streams, offline fan meetups, and collaborative content activities, digital nomads can be provided with social currency and emotional value, while also accumulating loyal assets for the long-term development of brands.

Furthermore, as digital nomads' expectations for the overall quality of shopping experiences continue to grow, greater attention and investment should be given to logistics efficiency, after-sales service guarantees, and personalized customization. Platforms and brand owners should consider the geographic mobility and plan variability of digital nomads in the fulfillment process, focus on the flexibility and traceability of logistics networks, and provide flexible delivery address management and transfer services. Currently, the market still has significant deficiencies in meeting the needs of digital nomads for personalized services, customization, and the convenience of cross-border returns and exchanges. Relevant enterprises should fully consider the special needs of digital nomad travelers, innovate in aspects such as modular product combinations, lightweight design, durability, environmentally friendly materials, and ease of repair, and strengthen the establishment of cross-border after-sales networks and cooperation mechanisms. They should provide seamless global warranties, convenient localized returns and exchanges, and explore innovative service models such as “product sojourning.” Logistics, insurance, and other related service providers can launch corresponding “Nomad Secure Shopping” plans, including services such as integrated luggage shipping, temporary storage, and duty-free customs clearance assistance, to provide solutions and a sense of security that align with the physiological mobility characteristics of digital nomads, thereby enhancing their satisfaction, willingness to recommend, and willingness to repurchase.

Furthermore, given that mobility is a typical characteristic of digital nomads, platforms and brands should focus on their social connection and emotional belonging needs. Limited by time fragmentation and spatial mobility, digital nomads seek convenience and recognition in online shopping participation. Ensuring fairness and security in the shopping process for digital nomads, enhancing their trust and satisfaction, not only meets the requirements of the trend of consumption upgrading but also aligns with principles such as inclusiveness and responsibility proposed in the “Agenda for Sustainable Consumption.” Therefore, considering that social recommendations and word-of-mouth more influence digital nomads' shopping decisions, online shopping platforms should pay special attention to community interaction functions and user-generated content systems in their design and operation, enhancing stickiness through building interest-based communities and establishing trust mechanisms, and strengthening personalized recommendation algorithms to create a precise and efficient shopping experience. Additionally, given the growing importance of product quality and consumption ethics among the digital nomad group, brands need to align with digital nomads' lifestyles and values, strengthen product traceability and disclosure of information on sustainability certifications, and offer product choices that align with their value propositions.

Third, and most importantly, this study introduces celebrity worship as a critical moderating mechanism. Unlike previous research that treated celebrity worship as a mere descriptive characteristic, we empirically demonstrate its conditional effects. For high-worship digital nomads, identity-building motivation becomes more powerful in driving satisfaction, while entertainment-seeking motivation paradoxically reduces satisfaction. This finding resolves apparent inconsistencies in prior livestream shopping research and highlights the necessity of measuring worship intensity rather than assuming its uniform effect.

Fourth, the motivational taxonomy identified in this study offers concrete leverage for the consumer-facing digitalization of China's characteristic forest-fruit industries, including those of Southern Xinjiang such as Korla pears, Xinjiang jujubes, and walnuts. The “identity pursuit” and “comprehensive recognition” segments, which are female-dominated, exhibit high repurchase rates, and are most strongly moderated by celebrity worship, constitute the most actionable target audiences for livestream promotion of geographically indicated agricultural products. Consistent with prior evidence that narratives of rural revitalization, ecological sustainability, and agricultural heritage amplify consumer emotional resonance and perceived credibility in agricultural livestream contexts (Jin and Zhang, 2025; Dong et al., 2022; Duan, 2023), content strategies for these segments should foreground cultural provenance, producer narratives, and sustainability credentials rather than price-led promotion. The “practical convenience” segment, by contrast, responds more readily to logistics transparency, information quality, and cold-chain fulfillment signals, which aligns directly with ongoing supply-chain digitalization investments in characteristic-industry regions and with the system-level dynamics described in recent agricultural livestream e-commerce ecosystem research (Zhao, 2025; Huo et al., 2025). Notably, the negative moderating effect of celebrity worship on entertainment-driven satisfaction suggests that livestream selling of characteristic forest-fruit products should avoid over-entertainment framing and instead anchor content in credibility, origin traceability, and community participation. Taken together, these findings supply a consumer-side evidence base that complements the predominantly supply-side focus of existing forest-fruit digital transformation research, and provides project-level guidance for Southern Xinjiang's forest-fruit digital transformation agenda by identifying which consumer segments are most responsive to which livestream content strategies.

Finally, to address the internal differences in the motivational types of digital nomad shoppers, platforms and brands can adopt the following strategies: (1) For socially interactive digital nomads, shopping scenarios with strong interactivity should be designed. Activities such as livestreaming voice chats, comment interactions, virtual communities, and offline fan gatherings can create a more immersive social experience and a sense of belonging, highlighting the community atmosphere and emotional value to enhance repurchase and recommendation intentions among online shoppers, fully unleashing their potential for social dissemination. (2) For practically convenience-oriented digital nomads, optimization of supply chain and logistics systems should be strengthened. By highlighting efficiency gains and cost-effectiveness, along with mobile experience and cross-platform collaboration, intelligent recommendations and one-stop services should be used to stimulate the latent purchasing power and loyalty of this group in the context of fragmented consumption. (3) For identity-seeking groups and comprehensive identity-seeking groups, brands should be more consistent and in-depth in conveying values and shaping brand stories. They should further strengthen community culture and brand loyalty around identity and self-expression, enhancing consumers' emotional attachment and recommendation intentions.

### 5.3 Limitations and future research directions

First, it should be noted that, due to practical limitations of online questionnaire surveys, digital nomads may face issues such as uneven geographical distribution, ambiguous occupational definitions, and difficulties in contact due to high mobility. These factors lead to biases in the population actually reached by the study, resulting in a sample that is concentrated among relatively younger digital nomads with higher educational attainment and income. Secondly, amid the deep integration of social media and e-commerce, which enhances the shopping experience and community cohesion among digital nomads, this study focuses on digital nomads' shopping motivations. It links “influencer-driven consumption” with “digital nomads” for integrated consideration, exploring their intrinsic motivations and behavioral characteristics. The aim is to fill a gap in related research and to attract more attention and discussion from academia and industry to the diversity and complexity of digital nomads' consumption behaviors. Future research can build on this to further explore the behaviors and psychology of digital nomads seeking identity recognition and community belonging across different cultural contexts, thereby expanding relevant theoretical models and practical applications.

Third, the present sample is drawn from coastal and central Chinese provinces and does not directly include consumers in Southern Xinjiang, nor does it explicitly measure purchases of characteristic forest-fruit products. Future work under this research program will extend the present motivational framework to a dedicated sample of livestream consumers of Southern Xinjiang forest-fruit products (for example, Korla pears, walnuts, and Xinjiang jujubes), testing whether the identified segment structure and the moderating role of celebrity worship replicate in that specific product and regional context, and thereby providing a more direct consumer-side evidence base for the digital transformation of characteristic forest-fruit industries.

## Data Availability

The raw data supporting the conclusions of this article will be made available by the authors, without undue reservation.

## References

[B1] Almanasreh E. Moles R. Chen T. F. (2019). Evaluation of methods used for estimating content validity. Res. Social Admin. Pharm. 15, 214–221. doi: 10.1016/j.sapharm.2018.03.066 29606610

[B2] Andino-Frydman A. (2023). “Who is a digital nomad? The evolving identities of the new nomadic workforce,” in Ethnographies of Work, Vol. 35, eds. R. Delbridge, M. Helfen, A. Pekarek, and G. Purser (Bingley: Emerald Publishing Limited), 95–137. doi: 10.1108/S0277-283320230000035005

[B3] Atanasova A. (2021). Digital nomadism as a critique of modern life: the role of consumption (Doctoral dissertation). Royal Holloway, University of London, London, United Kingdom.

[B4] Babin B. J. Darden W. R. Griffin M. (1994). Work and/or fun: measuring hedonic and utilitarian shopping value. J. Consumer Res. 20, 644–656. doi: 10.1086/209376

[B5] Bahri M. T. (2024). Evidence of the digital nomad phenomenon: from “Reinventing” migration theory to destination countries readiness. Heliyon 10, 56–78. doi: 10.1016/j.heliyon.2024.e36655 PMC11387329 39263067

[B6] Bardhi F. Eckhardt G. M. (2017). Liquid consumption. J. Consum. Res. 44, 582–597. doi: 10.1093/jcr/ucx050

[B7] Bartsch A. (2012). Emotional gratification in entertainment experience. Why viewers of movies and television series find it rewarding to experience emotions. Media Psychol. 15, 267–302. doi: 10.1080/15213269.2012.693811

[B8] Bauman Z. (2000). Liquid Modernity. Cambridge: Polity Press.

[B9] Behrendt C. Nguyen Q. A. Rani U. (2019). Social protection systems and the future of work: ensuring social security for digital platform workers. Int. Soc. Secur. Rev. 72, 17–41. doi: 10.1111/issr.12212

[B10] Bonneau C. Aroles J. Estagnasié C. (2023). Romanticisation and monetisation of the digital nomad lifestyle: the role played by online narratives in shaping professional identity work. Organization 30, 65–88. doi: 10.1177/13505084221131638

[B11] Brooks S. K. (2021). FANatics: systematic literature review of factors associated with celebrity worship, and suggested directions for future research. Curr. Psychol. 40, 864–886. doi: 10.1007/s12144-018-9978-4

[B12] Cai J. Wohn D. Y. Mittal A. Sureshbabu D. (2018). “Utilitarian and hedonic motivations for live streaming shopping,” in Proceedings of the 2018 ACM International Conference on Interactive Experiences for TV and Online Video (TVX '18), eds. H. Ryu, J. Kim, T. Chambel, T. Bartindale, V. Vinayagamoorthy, and W. T. Ooi (New York, NY: ACM), 81–88. doi: 10.1145/3210825.3210837

[B13] Chen H. Dou Y. Xiao Y. (2023). Understanding consumer engagement in live streaming commerce: the role of interactivity and trust. Electronic Commerce Res. Appl. 59, 104–115. doi: 10.1016/j.elerap.2023.101266

[B14] Chevtaeva E. Denizci-Guillet B. (2021). Digital nomads' lifestyles and coworkation. J. Destination Market. Manage. 21, 115–126. doi: 10.1016/j.jdmm.2021.100633

[B15] Chiu C. M. Chang C. C. Cheng H. L. Fang Y. H. (2009). Determinants of customer repurchase intention in online shopping. Online Information Rev. 33, 761–784. doi: 10.1108/14684520910985710

[B16] Chiu C. M. Wang E. T. G. Fang Y. H. Huang H. Y. (2014). Understanding customers' repeat purchase intentions in B2C e-commerce: the roles of utilitarian value, hedonic value and perceived risk. Information Syst. J. 24, 85–114. doi: 10.1111/j.1365-2575.2012.00407.x

[B17] De Loryn B. (2022). Not necessarily a place: how mobile transnational online workers (digital nomads) construct and experience “home”. Global Netw. 22, 103–118. doi: 10.1111/glob.12333

[B18] Dixon J. A. Kelley E. (2007). Theory revision and redescription: complementary processes in knowledge acquisition. Curr. Dir. Psychol. Sci. 16, 111–115. doi: 10.1111/j.1467-8721.2007.00486.x

[B19] Dong X. Zhao H. Li T. (2022). The role of live-streaming e-commerce on consumers' purchasing intention regarding green agricultural products. Sustainability 14:4374. doi: 10.3390/su14074374

[B20] Duan S. (2023). Producing new farmers in Chinese rural live e-commerce: platformization, labor, and live e-commerce sellers in Huaiyang. Chin. J. Commun. 16, 324–342. doi: 10.1080/17544750.2023.2203939

[B21] Ehn K. Jorge A. Marques-Pita M. (2022). Digital nomads and the COVID-19 pandemic: narratives about relocation in a time of lockdowns and reduced mobility. Social Media Soc. 8, 2056–2076. doi: 10.1177/20563051221084958

[B22] Farag S. Schwanen T. Dijst M. Faber J. (2007). Shopping online and/or in-store? A structural equation model of the relationships between e-shopping and in-store shopping. Transport. Res. A Policy Pract. 41, 125–141. doi: 10.1016/j.tra.2006.02.003

[B23] Gu Z. Zhao X. Wu D. (2025). A model of shoppertainment live streaming. Manage. Sci. 71, 6816–6835. doi: 10.1287/mnsc.2023.01724

[B24] Gupta S. Jaiswal R. Gupta S. K. (2024). Digital nomads: a systematic literature review and future research agenda. Tour. Rev. 78, 56–67.

[B25] Hair J. F. Black W. C. Babin B. J. Anderson R. E. (2010). Multivariate Data Analysis, 7th Edn. Upper Saddle River, NJ: Pearson Education.

[B26] Hannonen O. (2020). In search of a digital nomad: defining the phenomenon. Information Technol. Tour. 22, 335–353. doi: 10.1007/s40558-020-00177-z

[B27] He Y. Liu Q. (2024). The excessive celebrity worship behavior questionnaire: Chinese scale development and validation. PLoS ONE 19:e0303683. doi: 10.1371/journal.pone.0303683 38776313 PMC11111060

[B28] Hennig-Thurau T. Gwinner K. P. Walsh G. Gremler D. D. (2004). Electronic word-of-mouth via consumer-opinion platforms: what motivates consumers to articulate themselves on the Internet? J. Interactive Market. 18, 38–52. doi: 10.1002/dir.10073

[B29] Ho H. C. Chan Y. C. (2022). Flourishing in the workplace: a one-year prospective study on the effects of perceived organizational support and psychological capital. Int. J. Environ. Res. Public Health 19:922. doi: 10.3390/ijerph19020922 35055747 PMC8775957

[B30] Horton D. Wohl R. R. (1956). Mass communication and para-social interaction: observations on intimacy at a distance. Psychiatry 19, 215–229. doi: 10.1080/00332747.1956.11023049 13359569

[B31] Huo D. Zhang X. Qiao L. Tang A. Wang Y. (2025). Live streaming economy and green consumer behavior of farming products: a psycholinguistic forecasting by using social computation. Technol. Forecast. Social Change 200:123531. doi: 10.1016/j.techfore.2024.123531

[B32] Irimiás A. (2023). “The young tourist and social media,” in The Youth Tourist: Motives, Experiences and Travel Behaviour, eds. A. Irimiás, M. Mitev, and A. Michalkó (Bingley: Emerald Publishing Limited), 63–81. doi: 10.1108/978-1-80455-147-920231005

[B33] Jin W. Zhang W. (2025). The impact of e-commerce live streaming on purchase intention for sustainable green agricultural products: a study in the context of agricultural tourism integration. Sustainability 17:6850. doi: 10.3390/su17156850

[B34] Jiwasiddi A. Schlagwein D. Cahalane M. Cecez-Kecmanovic D. Leong C. Ractham P. (2024). Digital nomadism as a new part of the visitor economy: the case of the ‘digital nomad capital' Chiang Mai. Information Syst. J. 34, 1–34. doi: 10.1111/isj.12496

[B35] Khan E. A. Quaddus M. (2015). Development and validation of a scale for measuring sustainability factors of informal microenterprises – a qualitative and quantitative approach. Entrepreneur. Res. J. 5, 347–372. doi: 10.1515/erj-2014-0017

[B36] Kozinets R. V. (1999). E-tribalized marketing: The strategic implications of virtual communities of consumption. Eur. Manage. J. 17, 252–264. doi: 10.1016/S0263-2373(99)00004-3

[B37] Li J. Zhong S. (2025). Understanding the well-being of digital nomads from the perspective of self-determination theory – the case of China. Information Technol. Tour. 27, 1229–1251. doi: 10.1007/s40558-025-00331-5

[B38] Lovinska L. Kaliuha Y. Kryshtopa I. Korytnik L. Umerova A. (2020). “Report on labour in human resources management system,” in III International Scientific Congress Society of Ambient Intelligence (Dordrecht: Atlantis Press). doi: 10.2991/aebmr.k.200318.011

[B39] Luo X. Lim W. M. Cheah J.-H. Lim X.-J. Dwivedi Y. K. (2025). Live streaming commerce: a review and research agenda. J. Comput. Information Syst. 65, 376–399. doi: 10.1080/08874417.2023.2290574

[B40] Malinen S. (2015). Understanding user participation in online communities: a systematic literature review of empirical studies. Comput. Human Behav. 46, 228–238. doi: 10.1016/j.chb.2015.01.004

[B41] Martin C. A. Turley L. W. (2004). Malls and consumption motivation: an exploratory examination of older Generation Y consumers. Int. J. Retail Distrib. Manage. 32, 464–475. doi: 10.1108/09590550410558608

[B42] McCutcheon L. E. Lange R. Houran J. (2002). Conceptualization and measurement of celebrity worship. Br. J. Psychol. 93, 67–87. doi: 10.1348/000712602162454 11839102

[B43] Miguel C. Lutz C. Perez-Vega R. Majetić F. (2025). “Alone on the road”: loneliness among digital nomads and the use of social media to foster personal relationships. Media Cult. Soc. 47, 546–566. doi: 10.1177/01634437241290087

[B44] Misra S. Stokols D. (2012). A typology of people–environment relationships in the Digital Age. Technol. Soc. 34, 311–325. doi: 10.1016/j.techsoc.2012.10.003

[B45] Najafabadi M. K. Mohamed A. Onn C. W. (2019). An impact of time and item influencer in collaborative filtering recommendations using graph-based model. Information Process. Manage. 56. doi: 10.1016/j.ipm.2018.12.007

[B46] Nash C. Jarrahi M. H. Sutherland W. Phillips G. (2018). “Digital nomads beyond the buzzword: defining digital nomadic work and use of digital technologies,” in Information in Contemporary Society. iConference 2018. Lecture Notes in Computer Science, Vol. 10766 (Springer), 207–217. doi: 10.1007/978-3-319-78105-1_25

[B47] Oliver R. L. (1980). A cognitive model of the antecedents and consequences of satisfaction decisions. J. Market. Res. 17, 460–469. doi: 10.1177/002224378001700405

[B48] Oliver R. L. (1999). Whence consumer loyalty? J. Market. 63, 33–44. doi: 10.2307/1252099

[B49] Prester J. Cecez-Kecmanovic D. Schlagwein D. (2023). Toward a theory of identity performance in unsettled digital work: the becoming of ‘digital nomads'. J. Information Technol. 38, 399–417. doi: 10.1177/02683962231196310

[B50] Qiu Y. (2025). Unveiling the digital luminaries: exploring the impact of the Chinese internet celebrity economy on consumer behavior. J. Knowl. Econ. 16, 3058–3080. doi: 10.1007/s13132-024-02020-w

[B51] Rani U. Furrer M. (2020). Digital labour platforms and new forms of flexible work in developing countries: algorithmic management of work and workers. Competition Change 25, 212–236. doi: 10.1177/1024529420905187

[B52] Rook D. W. Fisher R. J. (1995). Normative influences on impulsive buying behavior. J. Consum. Res. 22, 305–313. doi: 10.1086/209452

[B53] Sansone R. A. Sansone L. A. (2014). “I'm Your Number One Fan” – a clinical look at celebrity worship. Innov. Clin. Neurosci. 11, 39–41. 24653942 PMC3960781

[B54] Simeli I. Christou E. Chatzigeorgiou C. (2025). Digital nomads as unintentional influencers in destination branding: a multi-method study of ambient influence. J. Theor. Appl. Electronic Commerce Res. 20:340. doi: 10.3390/jtaer20040340

[B55] Sutherland W. Jarrahi M. H. (2018). The sharing economy and digital platforms: A review and research agenda. Int. J. Inform. Manage. 43, 328–341. doi: 10.1016/j.ijinfomgt.2018.07.004

[B56] Tajfel H. Turner J. C. (1979). “An integrative theory of intergroup conflict,” in The Social Psychology of Intergroup Relations, eds. W. G. Austin and S. Worchel (Monterey, CA: Brooks/Cole), 33–47.

[B57] Taylor J. C. Carr E. G. (1992). Severe problem behaviors related to social interaction: 1: attention seeking and social avoidance. Behav. Modif. 16, 305–335. doi: 10.1177/01454455920163002 1385701

[B58] Thompson B. Y. (2019). “‘I get my lovin‘on the run': digital nomads, constant travel, and nurturing romantic relationships,” in The Geographies of Digital Sexuality, eds. K. J. Harp, A. K. Johnson, and L. J. Wood (Singapore: Springer), 69–90. doi: 10.1007/978-981-13-6876-9_5

[B59] Voss K. E. Spangenberg E. R. Grohmann B. (2003). Measuring the hedonic and utilitarian dimensions of consumer attitude. J. Market. Res. 40, 310–320. doi: 10.1509/jmkr.40.3.310.19238

[B60] Wan Q. Chen J. Yu C. Lu M. Liu D. (2024). Optimal marketing strategies for live streaming rooms in livestream e-commerce. Electronic Commerce Res. 9, 1–34.

[B61] Wang Y. Lu Z. Cao P. Chu J. Wang H. Wattenhofer R. (2022). How live streaming changes shopping decisions in e-commerce: a study of live streaming commerce. SSRN Electronic J. doi: 10.2139/ssrn.3874121

[B62] Wilkesmann M. Bassyiouny M. (2025). From leisure to labor: how workations are reshaping hospitality and destination marketing in the era of New Work. J. Destinat. Market. Manage. 36:100991. doi: 10.1016/j.jdmm.2025.100991

[B63] Willment N. (2020). The travel blogger as digital nomad: (re-) imagining workplace performances of digital nomadism within travel blogging work. Information Technol. Tour. Manage. 22, 391–416. doi: 10.1007/s40558-020-00173-3

[B64] Wu C. Wilkes R. (2018). Local–national political trust patterns: why China is an exception. Int. Polit. Sci. Rev. 39, 436–454. doi: 10.1177/0192512116677587

[B65] Yu K. Hannam K. Gössling S. (2025). The sustainability paradox of digital nomadism: mobility, consumption, and socio-environmental engagement. Tour. Rev. 9, 1–20.

[B66] Zeithaml V. A. Berry L. L. Parasuraman A. (1996). The behavioral consequences of service quality. J. Mark. 60, 31–46. doi: 10.1177/002224299606000203

[B67] Zhang K. Jia R. Dong S. Yang J. Yang Q. Zhang L. (2025). Mapping the links between celebrity worship and subjective well-being in Chinese undergraduates via network analysis. Behav. Sci. 16:28. doi: 10.3390/bs16010028 41594969 PMC12837141

[B68] Zhao G. Y. (2025). Agricultural products live streaming e-commerce ecosystem dynamics research. Front. Sustain. Food Syst. 9:1685996. doi: 10.3389/fsufs.2025.1685996

[B69] Zhao S. Wu X. (2021). Motivations and consumption practices of fostered idol fans: a self-determination theory approach. J. Consum. Market. 38, 91–100. doi: 10.1108/JCM-08-2019-3370

[B70] Zsila Á. McCutcheon L. E. Demetrovics Z. (2018). The association of celebrity worship with problematic Internet use, maladaptive daydreaming, and desire for fame. J. Behav. Addict. 7, 654–664. doi: 10.1556/2006.7.2018.76 30221539 PMC6426373

